# Surface Hardness of Polished Dental Zirconia: Influence of Polishing and Yttria Content on Morphology, Phase Composition, and Microhardness

**DOI:** 10.3390/ma18143380

**Published:** 2025-07-18

**Authors:** Andrea Labetić, Teodoro Klaser, Željko Skoko, Marko Jakovac, Mark Žic

**Affiliations:** 1 School of Dental Medicine, University of Zagreb, Gundulićeva 5, 10000 Zagreb, Croatia; andrea.labetic@gmail.com; 2 Ruđer Bošković Institute, P.O. Box 180, 10000 Zagreb, Croatia; teodoro.klaser@irb.hr; 3 Department of Physics, Faculty of Science, University of Zagreb, Bijenička, c. 32, 10000 Zagreb, Croatia; zskoko@phy.hr; 4 Department of Fixed Prosthodontics, School of Dental Medicine, University of Zagreb, Gundulićeva 5, 10000 Zagreb, Croatia

**Keywords:** multilayer zirconia, microhardness, ZirCAD Prime, Cercon ht ML, Katana ZIRCONIA, ZirCAD LT, polishing, Y-TZP

## Abstract

This study examined the relationship between microhardness, morphology, and phase composition of dental yttria-stabilized tetragonal zirconia polycrystals (Y-TZP), which directly impact their long-term clinical performance and durability. The primary objective was to investigate the effects of yttria content and polishing on the surface properties and hardness of these materials. Samples from ZirCAD Prime, Cercon ht ML, ZIRCONIA YML, and ZirCAD LT were analyzed using Vickers hardness testing, Powder X-ray Diffraction (PXRD), and Scanning Electron Microscopy (SEM). SEM analysis revealed a gradual increase in grain size and porosity with higher yttria content in unpolished samples. Polishing resulted in a relatively uniform surface morphology with observable striations across all samples, subsequently leading to similar Vickers hardness values for all polished samples. PXRD and SEM analyses identified that these similar hardness values were likely due to the predominant monoclinic phase on the surface, induced by polishing. These findings underscore the significant influence of yttria content and polishing on Y-TZP microstructure and surface hardness, highlighting their critical role in the long-term success and clinical applicability of dental restorations.

## 1. Introduction

The advancement of Computer-Aided Design and Computer-Aided Manufacturing (CAD/CAM) technology has facilitated the clinical use of high-strength ceramic materials based on zirconia, with the addition of yttria and/or alumina to enhance their properties. Among these, yttria (Y^3+^)-stabilized tetragonal zirconia polycrystals (Y-TZP) have gained widespread use due to their enhanced mechanical performance and structural stability. Numerous dental materials enter the market each year, driven by demands for aesthetic appearance, monolithic design, and CAD/CAM compatibility [1,2,3,4]. Y-TZP ceramics are particularly valued for their biocompatibility and excellent mechanical, chemical, and optical properties that closely resemble those of natural teeth [5,6]. As a result, they are widely preferred for prosthetic rehabilitations due to their strength, aesthetics, and clinical reliability [7,8].

The terms “multilayered” and “multilayer” Y-TZP systems are used to describe a wide range of products that differ in composition. Some of these Y-TZP systems are designated as “multilayered” due to their polychromatic gradients, which create shade variations to enhance optical properties. These systems are designed to gradually increase translucency from the more opaque cervical region to the translucent incisal edge, mimicking the natural appearance of teeth. However, as translucency increases, mechanical properties often diminish [9,10,11]. Additionally, recent modifications to Y-TZP have increased the yttria content (to around 4–5 mol%), resulting in structures with a higher proportion of cubic polycrystals (approximately 50%) and greater light transmission [12,13,14]. Therefore, the properties of different Y-TZP materials must be continually investigated and clarified.

Although a variety of dental Y-TZP materials satisfy ISO standards, mechanical testing remains essential, as clinical performance is influenced by factors such as manufacturing, storage, and handling [15]. These standards do not guarantee optimal clinical outcomes. Therefore, it is essential to independently evaluate the mechanical properties of dental Y-TZP materials through rigorous testing to ensure their suitability for clinical use. Mechanical testing provides valuable information on strength, durability, and wear resistance, which are crucial for long-term clinical success [16].

Furthermore, the Vickers hardness testing method can be used to determine material hardness at the microscale [17]. Before testing, Y-TZP materials require polishing, which is a critical step that ensures a smooth surface for accurate indentation. However, the polishing process itself may induce morphological changes and trigger a phase transformation from the tetragonal to the monoclinic phase, potentially affecting the Vickers hardness measurements.

Of the various hardness testing methods, the Vickers test is useful for evaluating a material’s resistance to permanent indentation and abrasive wear. For example, 4Y-TZP and 5Y-TZP exhibit higher Vickers hardness values (12.2–12.9 GPa) compared to natural tooth enamel (3–6 GPa). Nanoindentation with a Berkovich indenter provides an additional method for assessing hardness at the nanoscale. Enamel hardness values reported in the literature vary depending on the measurement scale and testing technique [18,19,20,21].

While Vickers hardness testing provides valuable microscale data, correlating these results with the phase composition of Y-TZP materials is of particular interest. Powder X-ray Diffraction (PXRD) is a widely accepted technique for characterizing crystalline phases. In addition, Scanning Electron Microscopy (SEM) is vital for analyzing morphological changes, particularly those influenced by Y^3+^ addition and surface polishing [17,22]. Therefore, the combined application of Vickers hardness testing, PXRD, and SEM is crucial for a comprehensive understanding of how morphology and phase composition influence the microhardness of Y-TZP materials [23,24].

This study aimed to systematically and precisely investigate the impact of polishing on the surface hardness of dental Y-TZP ceramics, with a particular focus on its effects on morphology and phase composition, both of which can ultimately influence mechanical performance and long-term clinical success. While polishing is a routine step in clinical preparation, it may induce structural changes, such as surface deformation or phase transformation, that alter these critical material properties. To explore this, we examined multiple Y-TZP systems using Vickers hardness testing, Powder X-ray Diffraction (PXRD), and Scanning Electron Microscopy (SEM) to correlate surface treatments with resulting material characteristics.

## 2. Materials and Methods

Three types of multilayered dental zirconia (Y-TZP) materials were selected for this study: (1) ZirCAD Prime (Ivoclar Vivadent AG; Schaan, Liechtenstein, I), (2) CERCON ht ML (Dentsply Sirona; Charlotte, NC, USA, S), and (3) Katana ZIRCONIA YML (Kuraray Noritake; Tokyo, Japan, K). Additionally, ZirCAD LT (Ivoclar Vivadent AG; Schaan, Liechtenstein, IK) was included as a control group (Table 1). Rectangular bar samples (16 mm × 2 mm × 2 mm) were milled from zirconia disks using a Programill PM7 milling machine (Ivoclar Vivadent AG) and sintered according to the manufacturer’s instructions in a Programat S1 1600 furnace (Ivoclar Vivadent-Technical).

For microhardness testing, the bars were polished using a Komet 94003 (Komet Dental, Lemgo, Germany) ceramic polisher set, which includes medium (pink) and fine (gray) rubber polishers. According to the ISO 6507-1:2023 standard [25], only polished samples are eligible for testing. This polishing process simulates the work of a dental technician, and the final finishing is typically performed after occlusal adjustment in a dental office. Polishing was carried out using a handheld technical handpiece (Volvere i7, NSK; Nakanishi Inc., Kanuma, Tochigi, Japan) operating at 10,000 revolutions per minute. Initially, the surface was polished with the pink rubber polisher (94003 M, Komet; Komet Dental, Lemgo, Germany, for 30 s across the entire surface. This was followed by polishing with the gray rubber polisher (94003 F, Komet; Komet Dental, Lemgo, Germany).

The multilayered bar samples, excluding ZirCAD LT (IK), exhibit an Y^3+^ gradient throughout their layers (Table 2). For this investigation, three representative layers were specifically chosen for evaluation: the incisal layer (5Y-TZP), the cervical layer (3Y-TZP), and the transit layer (3Y/5Y-TZP) (Table 2). These selected layers closely replicate the natural tooth gradient in terms of composition, thereby exhibiting varying mechanical properties and translucency. The microhardness and morphology of these samples were subsequently assessed using Vickers microhardness testing and Scanning Electron Microscopy (SEM), respectively.

**Figure 1:**
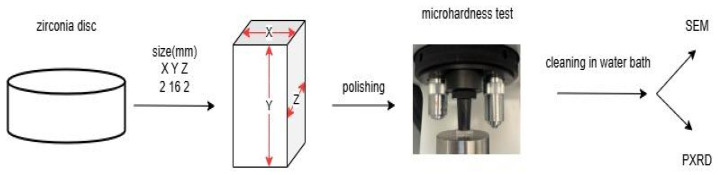
Preparation of polished Y-TZP bars with a Y^3+^ gradient from zirconia disk for Vickers measurements and further analyses. Prepared bar dimensions are *x* = 2 mm, *y* = 16 mm, and *z* = 2 mm, respectively. The indentations were placed on the XY plane. The yttria content gradually changes along the Y direction. Note: ZirCAD LT is not multilayered Y-TZP according to the manufacturer.

The Vickers microhardness of the specimen surfaces was measured using a digital microhardness tester (CSV-10; ESI Prüftechnik GmbH, Wendlingen, Germany) under a load of 1.96 N (HV 0.2) and a dwell time of 15 s. Following manual delineation of the indentation outlines, Vickers microhardness was calculated by the instrument software according to the equation VHN = 0.1891 × *F*/*d*^2^, where VHN denotes the Vickers hardness, *F* is the applied load (N), and *d* is the mean diagonal length of the indentation (mm). For each sample, five indentations were performed on each of the three layers of interest (incisal, cervical, and transit). The mean value of the five measurements was calculated and used as the statistical unit (Figure 1).

Ten bar samples were tested for each material type (Table 1). Vickers microhardness was evaluated at three distinct layers on each sample. Because the data did not significantly violate normality assumptions, a two-way ANOVA was performed to compare microhardness values among the four materials and across the three layers (5Y, 3Y/5Y, and 3Y). Due to significant interactions between material type and layer, separate one-way ANOVA tests were conducted to identify statistically significant differences within each material and layer. Following significant omnibus test results, Tukey’s post hoc adjustment was applied for pairwise comparisons. Statistical analyses were performed using SPSS software, version 26 (IBM, Armonk, NY, USA), with a significance level of 0.05 for all tests.

To comprehensively characterize the surface morphology of both unpolished and polished specimens, a Thermo Fisher Axia Chemi Scanning Electron Microscope (SEM) (Waltham, MA, USA) was utilized. Following microhardness testing, the samples were cleaned in a water bath, sputter-coated with gold to enhance conductivity, and mounted onto SEM stubs using double-sided carbon tape. SEM imaging was performed at 10 kV, with images captured at various magnifications to reveal surface features and, where discernible, the grain structure. Representative images of the specimens were displayed at 10,000× magnification.

Phase analysis of all four material types was conducted using Powder X-ray Diffraction (PXRD), employing a Bruker D8 Discover diffractometer (Karlsruhe, Germany) equipped with a LYNXEYE XE-T detector. Data were collected in Bragg–Brentano geometry (1D) using CuKα radiation (λ = 1.54 Å). The angular 2θ range was set from 10° to 70°, with a step size of 0.02° and a measuring time of 27 s per step. Rietveld refinement was performed using the HighScore Plus software (version 3.0, Malvern Panalytical, Almelo, The Netherlands). As a starting crystal structure model for refinement, we used cif no. 53998-ICSD for c-ZrO_2_, 185125-ICDS for m-ZrO_2_, and 164862-ICDS for t-ZrO_2_ (Inorganic Crystal Structure Database).

Grain size analysis from SEM images was performed using ImageJ (version 1.54p), an open-source image processing software developed in Java. Inspired by NIH Image, ImageJ is compatible with Windows, macOS, and Linux operating systems, and requires a Java Virtual Machine (version 1.4 or later). The software was accessed via the official ImageJ website (https://imagej.net/ij/). 

## 3. Results and Discussion

### 3.1. Microhardness Evaluation of Bar Samples Using Vickers Hardness Number (VHN)

**Figure 2:**
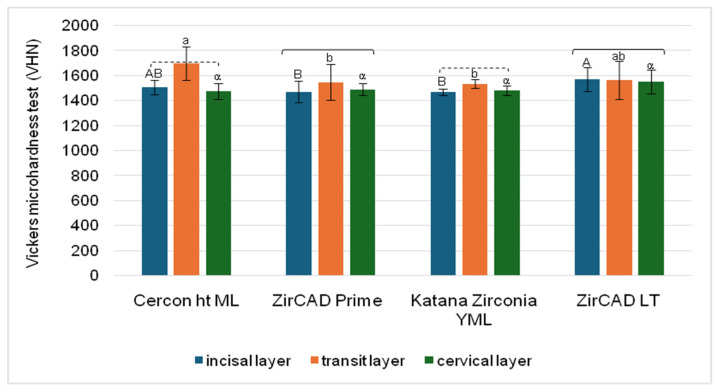
Bar plot showing Vickers microhardness values for four different materials across three positions (i.e., layers). Statistical comparisons are represented as statistically homogeneous groups. Statistically similar values (*p* > 0.05) between materials are indicated by identical letters: uppercase for the incisal layer, lowercase for the transit layer, and Greek letters for the cervical layer. Square brackets denote statistically similar values (*p* > 0.05) between layers within each material. Dashed square brackets indicate that the incisal and cervical layers were statistically similar (*p* > 0.05), but both were significantly different from the transit layer.

Microhardness analysis of the bar samples yielded values ranging from 1470.0 ± 27.7 VHN (lowest) to 1701.3 ± 133.5 VHN (highest) (Figure 2). For ZirCAD Prime and ZirCAD LT, all three layers exhibited statistically similar microhardness values. In contrast, CERCON ht ML and Katana ZIRCONIA YML showed statistically similar microhardness in the incisal and cervical layers, while their transition layers (3Y/5Y) exhibited significantly higher microhardness compared to both the incisal and cervical layers (*p* < 0.001 for both materials).

In statistical comparisons among materials, the results varied depending on the tested layer. For the incisal layer, ZirCAD LT exhibited significantly higher microhardness than both ZirCAD Prime and Katana ZIRCONIA YML (*p* = 0.048 and 0.025, respectively). In the transition layer (3Y/5Y), CERCON ht ML showed significantly higher microhardness than ZirCAD Prime and Katana ZIRCONIA YML (*p* = 0.020 and 0.019, respectively). In contrast, no statistically significant differences were observed among the four materials in the cervical layer.

Notably, the 3Y/5Y TZP transition layer in CERCON ht ML and Katana ZIRCONIA YML (orange bars) exhibited the highest hardness values, which is a striking observation. These findings suggest that the polishing process may have a particularly variable impact on the different compositional layers (Table 2) within these materials (Table 1). Therefore, further investigation into the factors influencing material hardness, especially the effect of polishing on individual layers, is warranted.

A narrow range of microhardness values was observed, particularly for the multilayer specimens (Figure 2), indicating consistent resistance to plastic deformation across all tested materials. This finding aligns with observations reported in the literature. For example, Zhang et al. [12] found no significant difference in microhardness between 5Y-TZP and various types of 3Y-TZP samples. Similarly, our study showed comparable microhardness values across all groups, regardless of production technique or orientation [26]. However, the values obtained in our study (Figure 2) were notably higher than those reported in previous research [27,28].

Next, these discrepancies may be attributed to several factors, including differences in measurement units, modifications in binder and zirconia content, or variations in production techniques. Additionally, the use of translucent zirconia, which typically contains 4.5 mol% to 6.0 mol% yttria and influences mechanical properties, may also contribute. Branco et al. and Harrer et al. similarly reported lower hardness values for additively manufactured (AM) specimens, regardless of measurement direction or printing orientation [5,29]. Therefore, careful consideration of specific experimental parameters and material properties is essential when comparing results across studies.

The observed disparities could be attributed to variations in (a) material composition (e.g., yttria content or other dopants), (b) manufacturing processes (e.g., sintering parameters or fabrication techniques such as milling versus 3D printing), and (c) testing conditions (e.g., polishing, applied load, dwell time, or measurement methodology) [30,31]. Notably, grain size also significantly influences microhardness; smaller grain sizes typically result in higher hardness values due to increased grain boundary resistance to deformation. Additionally, grain structure plays an essential role in the long-term performance of materials.

Therefore, future studies should aim to standardize these variables, particularly grain size, to facilitate more direct comparisons [32]. Additionally, it is important to acknowledge the limitations of the current study, including the aforementioned variations in material properties and manufacturing processes, which influenced the observed results. These limitations highlight the need for further investigation to fully understand the complex interplay of these factors. To explore the relationship between grain size and microhardness, and to visualize the microstructural features contributing to hardness variations, scanning electron microscopy (SEM) analysis was conducted, as detailed in the following section.

### 3.2. SEM Analysis

#### 3.2.1. Microhardness Indentation Imaging and Analysis

**Figure 3:**
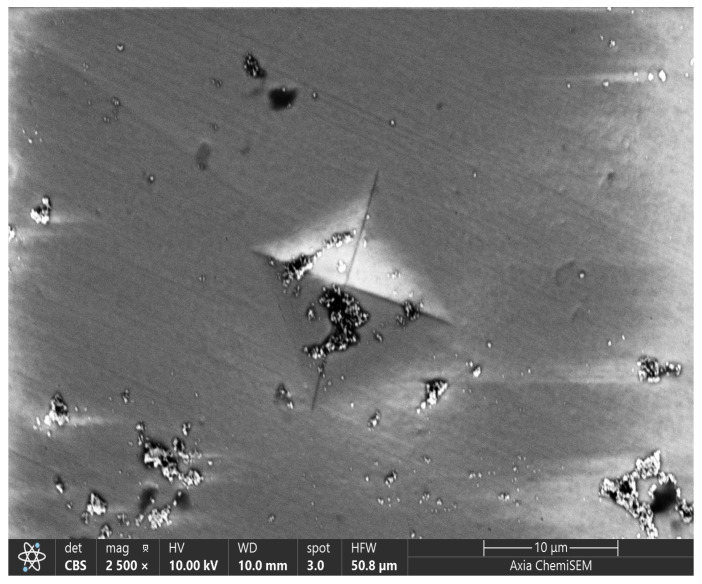
Microhardness indentation SEM image (10,000×)-monolithic CAD/CAM material ZirCAD Prime specimen.

Figure 3 shows the SEM image (10,000× magnification) of a Vickers pyramid indentation on the ZirCAD Prime bar sample. The indentation displays clear, sharp edges, indicating the material’s high hardness. The notable absence of significant material pile-up around the indentation is characteristic of brittle materials with limited plasticity [9]. This inherent brittleness of zirconia is counteracted by its ability to undergo stress-induced phase transformation, known as transformation toughening, which provides a crucial level of fracture resistance [33]. This ‘pseudo-plasticity’ is desirable for dental applications, enabling zirconia restorations to withstand the forces in the oral cavity. Scattered dark regions and small particles are visible, suggesting potential surface impurities or microstructural defects [32].

#### 3.2.2. SEM Imaging of Unpolished and Polished ZirCAD Prime (I) Bar Samples

**Figure 4:**
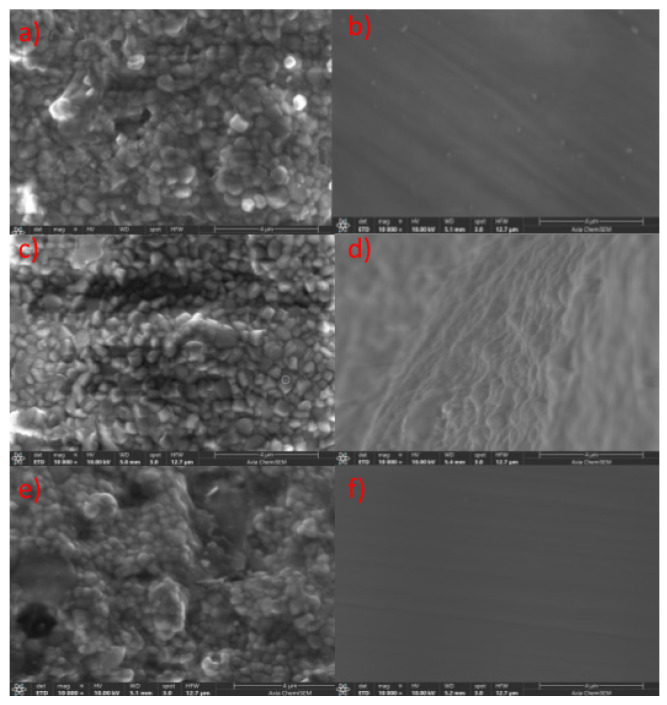
SEM images (10,000×) of ZirCAD Prime specimens taken at different positions before and after polishing. Images (**a**,**c**,**e**) show unpolished 3Y, 3Y/5Y, and 5Y-TPZ samples, whereas images (**b**,**d**,**f**) show polished 3Y, 3Y/5Y, and 5Y-TPZ samples.

Ongoing Figure 4 presents SEM images (10,000× magnification) of both unpolished and polished surfaces of the 3Y-TZP, 3Y/5Y-TZP, and 5Y-TZP layers from the ZirCAD Prime bar sample. In the unpolished samples (Figure 4a,c,e), a gradual increase in grain size and changes in morphology are observed with increasing yttria content, progressing from the 3Y-TZP to the 5Y-TZP layer. Since the bar surfaces underwent identical milling, the observed more open or closed morphologies directly correlate with higher or lower yttria content and lower or higher microhardness values, respectively. This trend is consistent with previous research on the impact of Y^3+^ on Y-TZP material properties [6].

The polishing process resulted in a relatively uniform surface morphology across all layers (Figure 4b,d,f). However, distinct striations indicative of polishing were observed in Figure 4b,f but were less pronounced in Figure 4d. These variations suggest potential inconsistencies in the polishing procedure [34,35]. The potentially less effective polishing of the 3Y/5Y-TZP layer may have contributed to its higher Vickers hardness, as shown in Figure 2. Conversely, the observed similarity in the polished surface morphology of 3Y-TZP and 5Y-TZP may explain the comparable microhardness values obtained (Figure 2), a relationship that warrants further investigation.

#### 3.2.3. SEM Imaging of Unpolished and Polished CERCON ht ML (S) Bar Sample

**Figure 5:**
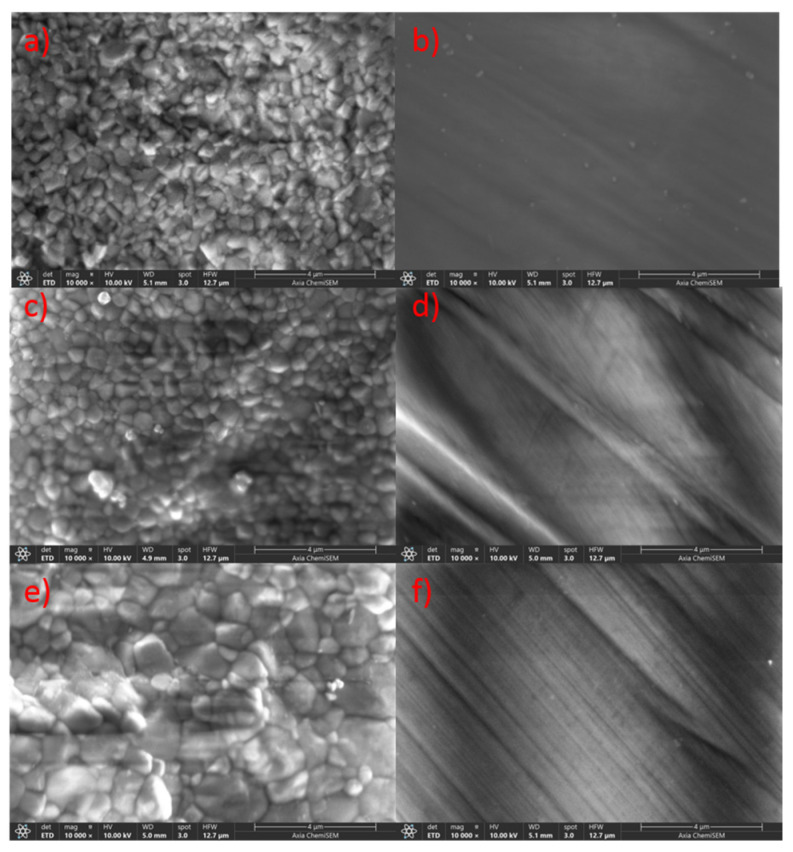
SEM images (10,000×) of CERCON ht ML specimens taken at different positions before and after polishing. Images (**a**,**c**,**e**) show unpolished 3Y, 3Y/5Y, and 5Y-TPZ samples, whereas images (**b**,**d**,**f**) show polished 3Y, 3Y/5Y, and 5Y-TPZ samples.

Figure 5a,c,e present SEM images (10,000× magnification) of the CERCON ht ML bar sample, showing unpolished surfaces of the 3Y-TZP, 3Y/5Y-TZP, and 5Y-TZP layers, respectively. Similarly to Figure 4a,c,e for ZirCAD Prime, the unpolished surfaces in Figure 5a,c,e display a gradual increase in grain size and a more open morphology with increasing yttria content. However, the 5Y-TZP layer in CERCON ht ML (Figure 5e) exhibits a significantly larger grain size compared to the 5Y-TZP layer of ZirCAD Prime (Figure 4e), potentially due to differences in phase composition [6].

The presence of striations (Figure 5b,d,f) reflects the effects of polishing. These striations were most evident on the 3Y/5Y-TZP and 5Y-TZP layers (Figure 5d,f), indicating a possible association with reduced surface hardness under the applied polishing conditions. Scanning Electron Microscopy further revealed an increase in grain size, especially in the 5Y-TZP layer, along with more prominent polishing striations in layers containing higher yttria content. Nevertheless, Powder X-ray Diffraction analysis is required to verify the relationship between yttria content and phase composition, which may provide additional insight into the observed hardness variations.

#### 3.2.4. SEM Imaging of Unpolished and Polished Katana ZIRCONIA YML (K) Bar Samples

**Figure 6:**
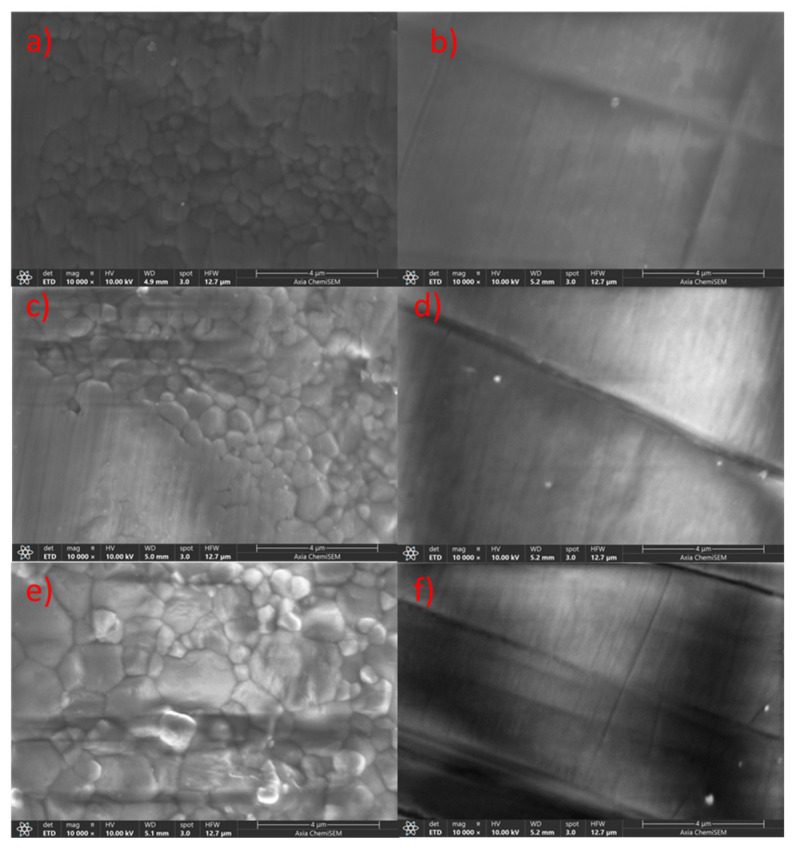
SEM images (10,000×) of Katana ZIRCONIA YML specimens taken at different positions before and after polishing. Images (**a**,**c**,**e**) show unpolished 3Y, 3Y/5Y, and 5Y-TPZ samples, whereas images (**b**,**d**,**f**) show polished 3Y, 3Y/5Y, and 5Y-TPZ samples.

Figure 6 presents SEM images (10,000× magnification) of the Katana ZIRCONIA YML bar sample, showing both unpolished (Figure 6a,c,e) and polished (Figure 6b,d,f) surfaces of the 3Y-TZP, 3Y/5Y-TZP, and 5Y-TZP layers, respectively. An increased yttria content corresponded with larger grain sizes, consistent with observations in Figure 4 and Figure 5. Notably, the grain size of the 5Y-TZP layer in this sample (Figure 6e) was the largest among all samples examined. The greatest grain size can be attributed to the highest content of the cubic phase.

The effect of polishing is shown in Figure 6b,d,f, where only striations are visible and no distinct grains can be detected. These striations are most pronounced in the 5Y-TZP layer (Figure 6f), potentially indicating the lowest surface hardness among the polished layers presented in Figure 4f, Figure 5f and Figure 6f. This observation correlates with the fact that the unpolished 5Y-TZP layer of this sample (Figure 6e) displayed the largest grain size compared to the other 5Y-TZP layers shown in Figure 4e and Figure 5e. These findings lead into the next section, where they will be further discussed. Overall, a higher yttrium ion content is associated with more pronounced striations and an increasing grain size gradient across the layers.

#### 3.2.5. SEM Imaging of Unpolished and Polished ZirCAD LT (IK) Bar Sample

**Figure 7:**
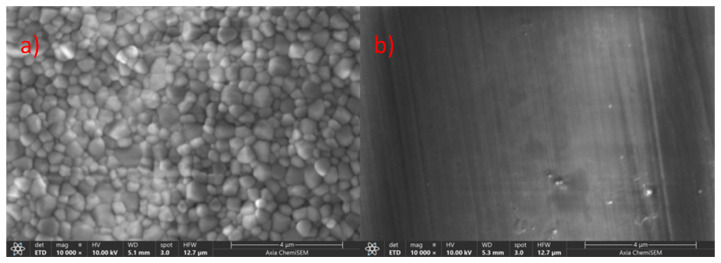
SEM images (10,000×) of ZirCAD LT specimens taken before and after polishing. Images (**a**,**b**) show an unpolished and polished 3Y-TPZ sample.

Figure 7 presents SEM images (10,000× magnification) of the ZirCAD LT (IK) bar sample, showing both unpolished (Figure 7a) and polished (Figure 7b) surfaces of 3Y-TZP. The unpolished sample exhibited a uniform grain size distribution, with the smallest 3Y-TZP grain size among the compared samples (Figure 4a, Figure 5a and Figure 6a). The polishing effect appeared minimal in this sample, which suggests it has the lowest yttria content and highest hardness among the materials tested. The observed variations in morphology and polishing effects across all samples underscore the need for PXRD analysis to correlate phase composition with these changes.

The SEM analysis revealed distinct morphological variations across all investigated bar samples. Notably, unpolished samples consistently exhibited a trend of increasing grain size and porosity with higher yttria content (Figure 4, Figure 5, Figure 6 and Figure 7), which correlated with lower microhardness values (Figure 2). Polishing, however, resulted in a relatively uniform surface morphology across all samples, characterized by striations. These striations were most pronounced in the 5Y-TZP layers, reflecting their larger grain sizes and higher yttria content (Figure 4f, Figure 5f and Figure 6f).

**Figure 8:**
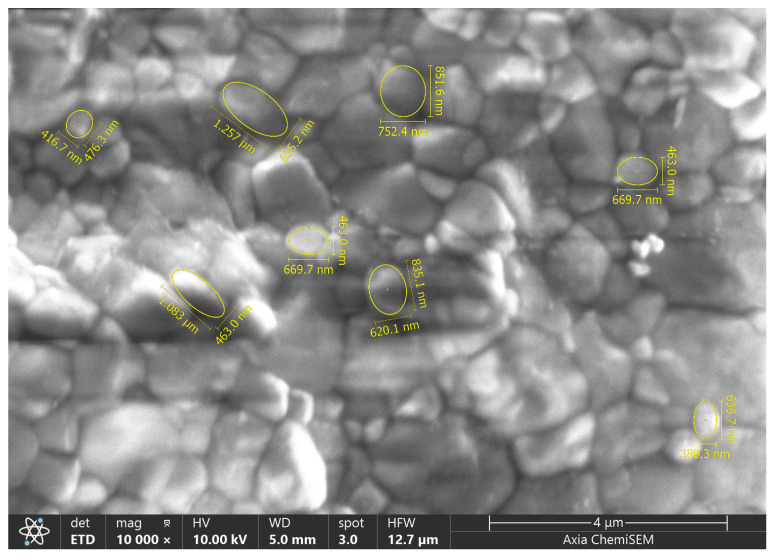
SEM image (10,000×) of the selected 5Y-TZP Sirona specimen. Grain size details were obtained through image analysis software.

Overall, these observations suggest that yttria content and polishing significantly influence both surface morphology and, consequently, the microhardness of these zirconia materials [36,37]. Grain size estimation, an example of which is presented in Figure 8, was conducted for all specimens, with the complete results detailed in Table 3. These observed differences in grain size further underscore the critical importance of microstructural control in optimizing the properties of dental zirconia, contributing to the ongoing research interest in these materials.

To ultimately correlate these morphological changes observed by SEM (Figure 4, Figure 5, Figure 6 and Figure 7) with specific phase compositions, Powder X-ray Diffraction (PXRD) analysis was performed. PXRD provided critical data on phase distribution and transformations, enabling a comprehensive understanding of the structure–property relationships in the investigated dental zirconia materials. This technique was crucial for determining the phase composition of the Y-TZP samples in this and in our previous [6] study.

### 3.3. Structural Investigations

**Figure 9:**
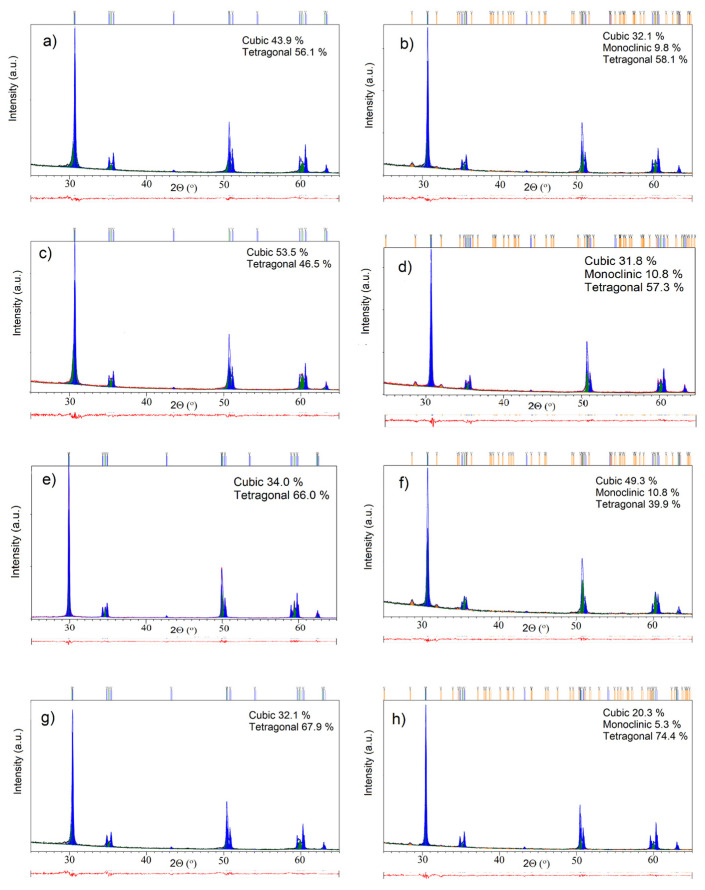
PXRD patterns of the investigated specimens. Unpolished specimens are marked as (**a**) CERCON ht ML, (**c**) ZirCAD Prime, (**e**) Katana ZIRCONIA YML, and (**g**) ZirCAD LT. Polished specimens are marked as (**b**) CERCON ht ML, (**d**) ZirCAD Prime, (**f**) Katana ZIRCONIA YML, and (**h**) ZirCAD LT.

Powder X-ray Diffraction (PXRD) analysis (Figure 9) of the investigated specimens (Table 1) revealed sharp, high-intensity diffraction peaks. This indicates that the specimens are highly crystalline, an expected outcome due to sintering at high temperatures (≈1500 °C). Additionally, the high crystallinity of the specimens is also consistent with observations from SEM images of unpolished samples (Figure 4, Figure 5, Figure 6 and Figure 7), which indicate well-defined grains characteristic of this type of Y-TZP material. By analyzing the positions and relative intensities of these peaks, the distinct crystalline phases present in the near-surface regions of the specimens were identified.

For a more quantitative analysis of the PXRD data, Rietveld analysis was conducted on the patterns depicted in Figure 9. This analysis yielded the phase compositions of the specimens, which are presented in Table 4. In the unpolished bar samples, the tetragonal phase was dominant in CERCON ht ML, Katana ZIRCONIA YML, and ZirCAD LT, while ZirCAD Prime was primarily composed of the cubic phase. None of the unpolished specimens showed the presence of the monoclinic phase.

However, following polishing, all samples exhibited an increase in the monoclinic phase, indicating a phase transformation induced by the polishing process [38]. Generally, polishing led to a reduction in the cubic phase and an increase in the tetragonal phase. Notably, the cubic phase decreased significantly in ZirCAD Prime and ZirCAD LT by approximately 46% and 37%, respectively, while the tetragonal phase increased by around 23% and 9%, respectively.

The phase analysis revealed that the monoclinic phase was present in all polished samples (Table 4). The emergence of the monoclinic phase at the surface may explain the similar Vickers hardness values observed across the polished samples [39]. These changes correlate with the consistent hardness results shown in Figure 2, suggesting a strong relationship between surface phase composition and hardness in these materials [28,39].

Although these findings provide useful information, there are some limitations to a full explanation of the results. Many dental materials used in this study are protected by patents and commercial confidentiality. Consequently, detailed information about their exact chemical composition, manufacturing processes, and proprietary formulations is not publicly available. This limits our ability to fully interpret data from methods like PXRD and SEM to better understand these materials.

## 4. Conclusions

This study comprehensively investigated the influence of yttria content and polishing on the microstructure, phase composition, and microhardness of various dental zirconia materials. Our findings confirm the highly crystalline nature of these specimens, a characteristic directly established by Powder X-ray Diffraction (PXRD) analysis and visually confirmed by Scanning Electron Microscopy (SEM) observations.

The sharp and well-defined edges of the Vickers pyramid indentations observed in the SEM image of the ZirCAD Prime sample, along with the absence of material pile-up, confirm the material’s ceramic nature and high hardness.

While SEM provided valuable surface-level insights, PXRD analysis revealed a dominant monoclinic phase on the polished surfaces, suggesting that polishing induced a phase transformation. This transformation likely explains the similar microhardness values observed across all tested materials, despite differences in their composition.

These findings highlight polishing’s significant impact, as it appears to homogenize surface microhardness and reduce the visible contrast between the polished material surfaces.

The high hardness values observed in this study are consistent with previous findings on zirconia ceramics and underscore the important roles of material composition, fabrication methods, and surface preparation, particularly polishing, in determining mechanical performance and clinical suitability.

Future research should aim to clarify the mechanisms underlying polishing-induced phase transformations, especially the formation of the monoclinic phase, and assess their implications for wear resistance, aging behavior, and long-term clinical durability.

## Figures and Tables

**Table 1:** Specifications of zirconia disk material specimens used in the study.

Specimen Designation	Manufacturer	Material	Multilayer Composition
I	Ivoclar Vivadent AG	ZirCAD Prime	3Y-TZP-5Y-TZP
S	Dentsply Sirona	CERCON ht ML	3Y-TZP-5Y-TZP
K	Kuraray Noritake	Katana ZIRCONIA YML	3Y-TZP-5Y-TZP
IK	Ivoclar Vivadent AG	ZirCAD LT	3Y-TZP

**Table 2:** Layer designations of the different multilayered Y-TZP bar specimens used in this study. Note: ZirCAD LT is not multilayered Y-TZP according to the manufacturer.

Layer Name	Designation
Incisal	5Y-TZP
Cervical	3Y-TZP
Transit layer	3Y/5Y-TZP

**Table 3:** The average grain size (µm) of specimens selected in this study.

Material	3Y-TZP	3Y/5Y-TZP	5Y-TZP
ZirCAD Prime	0.43	0.45	0.43
CERCON ht ML	0.49	0.45	0.54
Katana ZIRCONIA YML	0.48	0.51	0.53
ZirCAD LT	0.21	-	-

**Table 4:** Phase composition of the investigated bar samples as determined by Powder X-ray Diffraction (PXRD). Change (%) = ((Polished − Unpolished)/Unpolished) × 100%.

Sample	Phase	Polished	Unpolished	Change (%)
CERCON ht ML	c-ZrO_2_ (%)	32.1	43.9	−26.9
	t-ZrO_2_ (%)	58.1	56.1	3.6
	m-ZrO_2_ (%)	9.8	0	-
ZirCAD Prime	c-ZrO_2_ (%)	31.8	53.5	−40.6
	t-ZrO_2_ (%)	57.3	46.5	23.2
	m-ZrO_2_ (%)	10.8	0	-
Katana ZIRCONIA YML	c-ZrO_2_ (%)	49.3	34.0	45.0
	t-ZrO_2_ (%)	39.9	66.0	−39.6
	m-ZrO_2_ (%)	10.8	0	-
ZirCAD LT	c-ZrO_2_ (%)	20.3	32.1	−36.8
	t-ZrO_2_ (%)	74.4	67.9	9.6
	m-ZrO_2_ (%)	5.3	0	-

## Data Availability

The original contributions presented in this study are included in the article. Further inquiries can be directed to the corresponding authors.
